# Supplementary figures and images for: Phenotypic plasticity evolves at multiple biological levels in response to environmental predictability in a long-term experiment with a halotolerant microalga
Source: PLoS Biol. 2023 Mar 24;21(3):e3001895. doi: 10.1371/journal.pbio.3001895 (PMC10075460; doi:10.1371/journal.pbio.3001895)

**A.**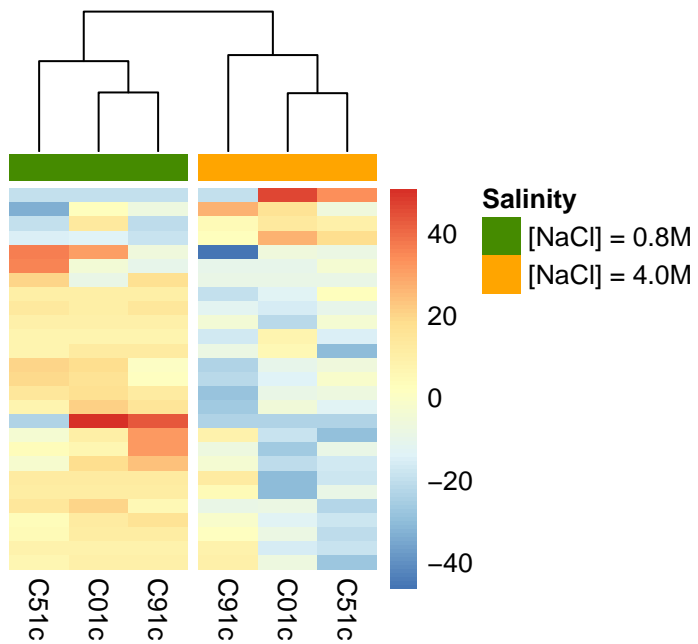**B.**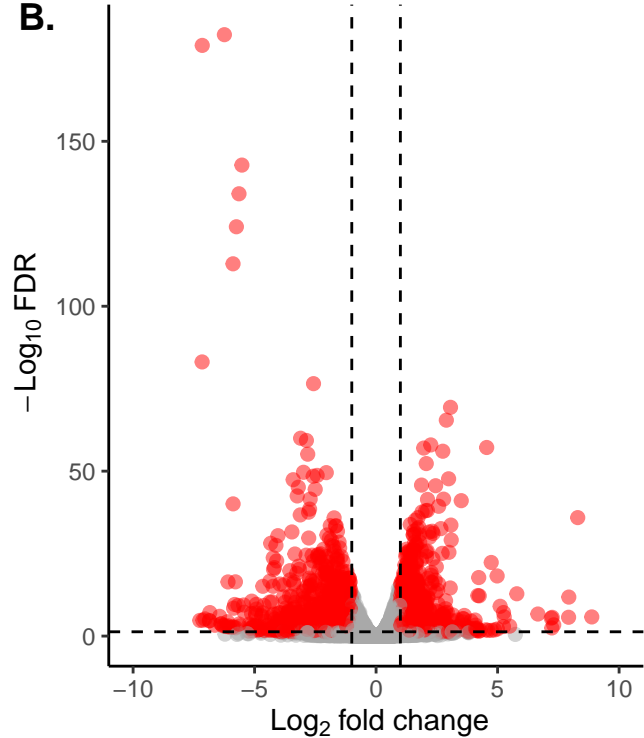

Supplement: S1 Fig — We founded 3 populations from single isolated cells from 3 evolved populations, following the protocol in Leung and colleagues [5]. As D. salina is haploid, a population founded from a single cell is expected to be isogenic. (A) Heat-maps of WGB-seq analysis for DMRs between salinities (n = 27). Each row represents a DMR, and column names are the population identity. Relative DNA methylation levels vary from blue (under-methylated) to red (over-methylated), as shown on the right-hand side of the heat-maps. Dendrograms on the top result from a hierarchical clustering analysis using the Euclidean distance of DNA methylation level among populations. (B) Volcano plot illustrating significant (for FDR < 0.05 and |Log2FC| > 1) and nonsignificant DE transcripts between salinities as red and gray points, respectively. Salinity effect was assessed by comparing three isogenic populations (i.e., found from a single cell). The raw data underlying this figure are available in the Figshare repository https://doi.org/10.6084/m9.figshare.21905670. DE, differentially expressed; DMR, differentially methylated region; WGB-seq, whole-genome bisulfite sequencing. (PDF) [file pbio.3001895.s003.pdf]
