# Supplementary material for: Phenotypic plasticity evolves at multiple biological levels in response to environmental predictability in a long-term experiment with a halotolerant microalga
Source: PLoS Biol. 2023 Mar 24;21(3):e3001895. doi: 10.1371/journal.pbio.3001895 (PMC10075460; doi:10.1371/journal.pbio.3001895)
Supplement: S2 Fig — Principal component analysis (PCA) of (A) DNA methylation and (B) gene expression levels among isogenic (open symbols) and experimental non-isogenic (filled symbols) populations. The raw data underlying this figure are available in the Figshare repository https://doi.org/10.6084/m9.figshare.21905670. (PDF) [file pbio.3001895.s004.pdf]

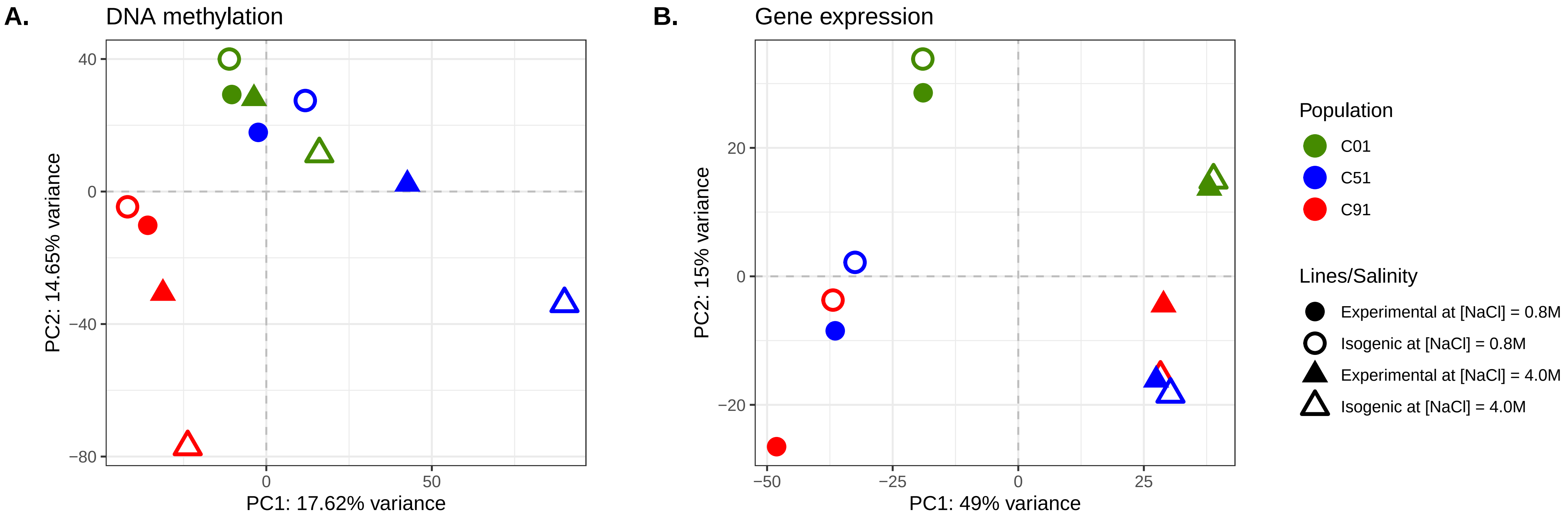

A.
DNA methylation
PC1: 17.62% variance
PC2: 14.65% variance
40
0
−40
−80
0
50
B.
Gene expression
PC1: 49% variance
PC2: 15% variance
20
0
−20
−50
−25
0
25
Population
C01
C51
C91
Lines/Salinity
Experimental at [NaCl] = 0.8M
Isogenic at [NaCl] = 0.8M
Experimental at [NaCl] = 4.0M
Isogenic at [NaCl] = 4.0M
