# Supplementary material for: Phenotypic plasticity evolves at multiple biological levels in response to environmental predictability in a long-term experiment with a halotolerant microalga
Source: PLoS Biol. 2023 Mar 24;21(3):e3001895. doi: 10.1371/journal.pbio.3001895 (PMC10075460; doi:10.1371/journal.pbio.3001895)
Supplement: Attachment — Submitted filename: Response_to_reviewer_PlosBiol2023_VF.pdf [file pbio.3001895.s005.pdf]

Montpellier, January 26th, 2023

Dear Dr. Roberts,

Please find enclosed a revision of our manuscript PBIOLOGY-D-22-02338R1 entitled: "The molecular basis of phenotypic plasticity evolves in response to environmental predictability" by Christelle Leung, Daphné Grulois, Leandro Quadrana and Luis-Miguel Chevin.

We are grateful to the editor and the reviewers for their interest and the positive feedback, as well as the useful suggestions for improvement of our manuscript. As you will see, we have considered all these comments carefully and revised the manuscript accordingly, as can be seen in our point-by-point responses below (where our responses appear in blue), and in a version of the manuscript with tracked changes.

We think that these changes, together with other minor edits and additions (in particular to the cited literature), have improved the clarity of our manuscript, so we hope that you will now find it suitable for publication in PLOS Biology.

Sincerely yours,

Christelle Leung, on behalf of all authors

EDITORS' COMMENTS

a) Please attend to the requests from the reviewers. The Academic Editor asked me to emphasise the following point, raised by reviewer #1: "... one additional bit of analysis has to do with repeatability. There were three replicates for each treatment. Were the same molecular responses seen in each?"
Please, see our response to reviewer #1.

b) Please provide a blurb, according to the instructions in the submission form.
We propose the following sentences as a blurb for our manuscript:
"Consistent evolution of reduced plasticity in less predictable environment was observed across three biological levels – epigenomics, transcriptomics, and cell morphology - , providing experimental evidence about the mechanisms of plasticity evolution across the genotype-phenotype map."

c) Please comply with our Data Policy; specifically, we need you to supply the numerical values underlying Figs 2AB, 3AB, 4, 5ABC, S1AB, either as a supplementary data file or as a permanent DOI'd deposition (e.g. Figshare, Dryad, Zenodo, etc.).
We have deposited the raw sequence data (RNA-seq and WGB-seq) used in this study in the NCBI's Sequence Read Archive (SRA) database under BioProject PRJNA736997 (https://www.ncbi.nlm.nih.gov/bioproject/?term=PRJNA736997). In addition, DNA methylation 100 bp tiles percentage values, raw RNA-seq read counts (i.e. transcript expression values), structural and functional gene annotations, and cell morphology data have been deposited in Figshare https://doi.org/10.6084/m9.figshare.21905670. The data will be made publicly available upon acceptance of the manuscript.

d) Please cite the location of the data clearly in all relevant main and supplementary Figure legends, e.g. "The data underlying this Figure can be found in S1 Data" or "The data underlying this Figure can be found in https://doi.org/XXXX"
We confirmed that we have specified the location of the data in all Figure legends.

REVIEWERS' COMMENTS:

Reviewer #1:
[identifies himself as Samuel Scheiner]

This manuscript reports on a deep dive into the molecular bases of an evolved response to an artificial selection experiment by phenotypic plasticity. In general the experiment is well designed and carried out. I have no complaints with what was done, although I do have one suggested additional bit of analysis.

However, I do have one complaint with the way that the work is being framed. According to the paper: "However, we still lack critical empirical evidence on the extent to which selection on phenotypic plasticity cascades down from higher phenotypic levels to their underlying molecular basis." We have decades of information that plasticity has a heritable genetic basis and that selection on plasticity can result in an evolutionary response. We even have a growing list of studies of the molecular bases of plasticity. So to say that we "lack critical empirical evidence" is incorrect.
What we do lack, and what this paper clearly explores, is the ways that different types of molecular changes respond to different intensities of selection for plasticity. The use of three different levels of predictability, combined with the detailed molecular and cellular measurements makes this a very valuable study. There is no need to oversell it.

We thank Dr. Scheiner for the positive comments, and we do agree with him that numerous studies have already demonstrated the genetic basis of plasticity. What we had attempted to emphasize was the lack of empirical evidence on how the hierarchical levels of molecular mechanisms underlying phenotypic plasticity (i.e. epigenetic changes and gene expression) evolve in response to selection for plasticity, as correctly mentioned by Dr Scheiner. We agree that this may not have been stated clearly enough, so we have reformulated the corresponding section in the abstract to avoid such misunderstanding, which now reads:

Lines 21-23: "*However, the extent to which patterns of evolution of plasticity for more integrated traits are mirrored by their underlying molecular mechanisms remains unclear, especially in response to well-characterized selective pressures exerted by environmental predictability*."

I do have one question about the results. For the analysis of DNA methylation, the text states that there was no rho-salinity interaction (line 110). But looking at Fig 2A, there looks like there is a pretty strong interaction (no salinity difference at low predictability and a large difference at predictability). Perhaps I am misunderstanding the figure, or there is a problem with low power as the total variation being explained is very small. Whatever the explanation, something needs to be said in the text as other readers are likely to have the same reaction as me. Or perhaps you can add confidence areas around the points.

We indeed failed to detect significant ρ^2 × salinity interaction due to very low variation being explained by this factor (adjusted R^2 = 0.30%, Line 113), but as pointed by Dr. Scheiner, Fig2A, which is a constrained ordination that maximizes variation in DNA methylation that can be explained by salinity and environmental predictability, actually suggests that lines from highly predictable environments displayed higher salinity differences than those from lowly predictable environments. Such results could be explained by more localized epigenetic differences, rather than a significant genome-wide epigenomic changes. We confirmed this hypothesis by investigating regional changes in DNA methylation at a finer scale, by considering non-overlapping 100 bp windows genome-wide, and observed that lines from low predictable environments actually displayed as little as three differentially methylated regions (DMRs) between salinities compared to 27 or 76 DMRs for lines from high and intermediate predictability (Fig. 2B).

We have explained these observations at lines 114-121:

"*Fig 2A, which is a constrained ordination that maximizes variation in DNA methylation that can be explained by salinity and environmental predictability, suggests that lines from highly predictable environments displayed higher salinity differences than those from lowly predictable environments. However, we did not detect a significant marginal effect of salinity (R^2_{adj} = 0.79%; P = 0.356) or ρ^2 × salinity interaction (R^2_{adj} = 0.30%; P = 0.562) on the overall DNA*

methylation pattern, suggesting the absence of overall DNA methylation changes in response to salinity or a lack of power to detect small variation being explained.

That we did not detect significant differentiation at the whole epigenome level does not preclude the existence of more localized epigenetic differences along the genome, so we also investigated regional changes in DNA methylation at a finer scale, by considering non-overlapping 100 bp windows, hereafter denoted Differentially Methylated Regions (DMRs).”

The one additional bit of analysis has to do with repeatability. There were three replicates for each treatment. Were the same molecular responses seen in each? In figures 2 and 3 the replicates appear to cluster closely, implying repeatability, but something should be said explicitly about this issue. If there were differences, were they coordinated across levels? (I will admit that I am not sure exactly how that could be analyzed or shown.) If you can add this, it would help deepen the entire story.

The different samples per evolutionary treatment we used are indeed biological replicates which enabled us to test for differences among evolutionary treatments. We observed that populations within the same conditions (i.e. salinity and evolutionary treatments) were more similar than those among conditions, and the significant DMRs and DE transcripts detected between salinity for each evolutionary treatments can be interpreted as concerted molecular (DNA methylation or gene expression, respectively) responses to changing salinity across samples of a given evolutionary treatment. We elaborated this point in the discussion section for clarification:
Lines 267-272:
“*Here, the significant DMRs and DE transcripts detected across evolutionary treatments can be interpreted as parallel evolution of DNA methylation and gene expression, respectively. Moreover, we not only found substantial plasticity at the levels of DNA methylation, gene expression, and cell morphology, but also that plasticity evolved in a consistent direction - and consistent with theoretical predictions - in response to environmental predictability across these levels.”*

Minor items

Lines 81-98: This material should be in Methods.
Given the structure of the manuscript required by the journal (Material and methods section at the end), we kept this section here to facilitate the comprehension of the results.

Lines 108-111: Besides just stating which terms were significant, it would helpful to add some sort of explanation about what each term means with respect to the main questions being posed. Or perhaps, this could be framed in the Introduction.
Thanks for this suggestion, we now write at the end of the first paragraph of the results (101-103):

“*At any of these levels, an effect of salinity is indicative of phenotypic plasticity, an effect of the evolutionary treatment* ρ^2 *denotes evolution, while* $\rho^2 \times$ *salinity interaction indicates evolution of plasticity.*”

Line 128: change: "total (Table S2). As [with]"
Corrected

Line 154: change "involved genes associated [with] cellular components"
Corrected

Line 157: change "[found] that"
Corrected

Line 158: insert "functions associated [with] different metabolic processes"
Corrected

Line 160 change "DNA methylation in cis was not associated"
Corrected

Line 168: change "regions were associated [with] significant DE transcripts"
Corrected

Line 177: "plasticity for each of them, measured" It is unclear what "them" refers to.
“them” referred to the three levels at which we assessed plasticity. We have now clarified this point:
Lines 190: “*we then quantified the overall degree of plasticity for DNA methylation, gene expression and cell morphology, measured as the Euclidean distance between low and high salinities*”

Line 179-181: As above, it would be useful to provide some sort of explanation of the meaning of this ordering.
Explanation of the meaning of the observed correlation is reported at the following sentence, i.e. now lines 195-198: “*Hence, populations that have experienced less predictable environments during experimental evolution have evolved reduced plasticity, not only for cell morphology (as shown by Leung, et al. [5]), but also for DNA methylation and gene expression, two important molecular mechanisms potentially underlying phenotypic plasticity*”

Lines 188-189: delete "We [] therefore exposed experimental"

Corrected

Line 197: "higher phenotype" I am not generally in favor of the notion of higher and lower in this context. Perhaps, "more integrated".
We have changed "*higher phenotype*" to "*more integrated phenotype*" throughout the manuscript.

Line 199: change "that this species [has] specific genes"
Corrected

Line 200: change "Salinity [for] gene"
Corrected

Line 204: change "involved genes associated [with]"
Corrected

Line 208: change "molecular machineries [for coping] consistently with"
Corrected

Line 216: insert "suggesting that [the] regulation of gene"
Done

Line 219: insert "DNA methylation measurement[s]"
Done

Line 220: insert "significant correlation[s] between DNA"
Done

Line 222: change "relying on [mechanisms other] than DNA"
Done

Line 228: change "could contribute to [fine-tuning] the"
Corrected

Line 235: insert "work has [e]stablished that the amount"
Corrected

Line 238: change punctuation "level above[,] for instance,"

Corrected

Line 247: change "across levels can [provide important]"
Corrected

Line 248: insert "the genotype-(environment)-phenotype [map]"
Corrected

Line 249: change "this can yield insights [about] the extent"
Corrected

Line 262: insert "For instance, expression [of] plasticity of a given gene"
Done

Line 286: insert and change "similar level[s] of morphological plasticity [were] found between"
Corrected

Line 288: I do not understand what is meant here. This is the first mention of any sort of negative autocorrelation.
Intermediate predictability (ρ^2 = 0.25) could be achieved through negative or positive autocorrelation (ρ = -0.5 or 0.5). Both autocorrelations were used in our experimental evolution experiment, as described in Leung et al. 2020. In the interest of accuracy and transparency, we reported the existence of these two autocorrelations values used to assess intermediate predictability (as we can read in, now, Lines 312-315), but we specified that only the ρ = -0.5 was used in the current study.

Line 290: insert "the protocol descri[b]ed in Leung"
Corrected

Line 318: change "population affect[ed] the observed plasticity"
Corrected

Line 323: delete "plasticity assay [] described above"
Corrected

Line 406: change "data was [replicated here], rather than"
Corrected

Reviewer #2:
[identifies himself as Carl Schlichting]

This study examines patterns of methylation and transcription for 9 lines of Dunaliella subjected to Randomly fluctuating salinity for 500 generations; three regimes produced differences in autocorrelation (i.e., predictability of 0.00, 0.25 and 0.81. Lines were subsequently exposed to 0.8M or 4.0M salinity to assess plasticity and gene expression.
Results suggest that lack of predictability has the predicted effect of reducing plasticity.
Results of analysis of methylation and transcription patterns are similar, but not congruent: DMRs are only affected by evolutionary history; DEs affected mostly by salinity level, with a significant EH*salinity interaction.
A very solid study, with robust methods and analyses.

We thank Dr. Schlichting for the positive evaluation of our work.

Comments
The authors state (L 141) that there is "great overlap among DE transcripts identified between salinities for the different evolved lines indicating that the plastic response to salinity largely involved transcriptional regulation of a common pool of genes". By my calculations this is only about 15% - there were 70% more DEs specific to EHs than those found in all EHs. Even if you add in all overlaps the numbers are almost the same.

We do agree with reviewer #2 that the adjective "great" might not be appropriate with respect to the total number of DE transcripts between salinities. What we wanted to emphasize was the difference with DNA methylation, for which we did not detect any overlap among DMRs, while we detected much more similar DE transcripts between salinities for all evolutionary treatments. We thus modified the sentence, which now reads:
Lines 151-154: "*However, in contrast to what we found for DNA methylation, we observed substantial overlap among DE transcripts identified between salinities for the different evolved lines (Fig. 3B), indicating that the plastic response to salinity largely involved transcriptional regulation of a common pool of genes, regardless of their evolutionary trajectory. Nonetheless, we still detected some* ρ^2*-specific DE transcripts between salinities (Fig. 3B).*"

L 155 - No GO term enrichment with FDR ≤ 0.1 was detected for DE transcripts among evolutionary treatments - any ideas about why this is?

We think that the lack of enriched GO terms is because of low power for annotation analysis, as only ~ 30% of all identified transcripts were successfully assigned to a given GO terms (Leung et al. 2020). This was specified in the discussion section, at lines 2016-217.

L 215 - "we failed to detect a correlation between DMRs and the expression of proximal genes"
I couldn't find this documented in the results anywhere - please include

This sentence actually refers to results described in lines 173-184.

L 323 - Did you compare isogenic lines with experimental lines?

Each isogenic line was founded by isolating a single cell from the corresponding experimental line. The purpose of this manipulation was to minimize the possibility that observed changes in DNA methylation and gene expression across salinity were caused by rapid selection response in the (polymorphic) evolved lines, by checking that we observed similar responses in lines with no (or extremely little) genetic variation. To further validate this approach, we have now also verified, using partial RDA, that isogenic lines did not significantly differ from corresponding experimental lines, for both DNA methylation (P = 0.423) and gene expression (P = 0.520).

We have added this new result in lines 161-163 of the new version of the manuscript: "*Furthermore, we did not detect any significant differences in either DNA methylation (P = 0.423) or Gene expression (P = 0.520) between isogenic lines and the experimental populations they originated from (Fig. S2).*"

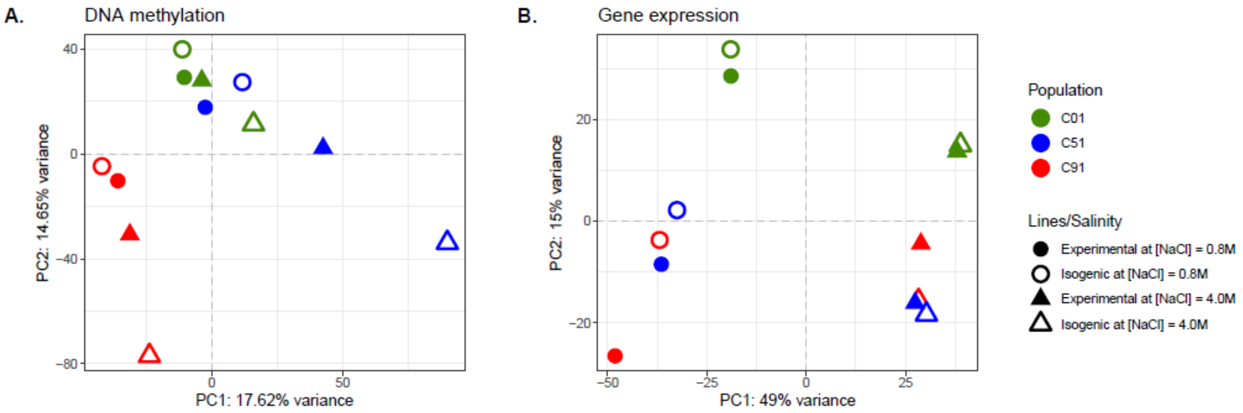

Fig. S2. Comparison of isogenic and experimental populations. Principal component analysis (PCA) of (A) DNA methylation and (B) gene expression levels among isogenic (open symbols) and experimental non-isogenic (filled symbols) populations. The raw data underlying this figure are available in the Figshare repository https://doi.org/10.6084/m9.figshare.21905670.

Para at L 386 - I couldn't follow the description of the RDA analysis. RDA/PCA typically doesn't partition effects into explanatory variables. Did you use the RDA output as an input into ANOVA?

We do agree with Dr. Schlichting that principal component analysis (PCA) does not partition effects into explanatory variables, as it is an unconstrained analysis. However, we performed the variation partition using redundancy analysis (RDA), as proposed by Borcard et al. 1992. More specifically, the use of partial RDA enabled us to calculate the effects of salinity, ρ^2 and $\rho^2 \times$ *salinity* on DNA methylation, gene expression or cell morphology.
We specified "partial RDA" in the revised version of the manuscript.

L 288, garbled
A couple of instances of Euclidian rather than Euclidean

Thank you for pointing out this misspelling, which is now corrected.
